# Substance Use, Mental Disorders and Physical Health of Caribbeans at-Home Compared to Those Residing in the United States

**DOI:** 10.3390/ijerph120100710

**Published:** 2015-01-13

**Authors:** Krim K. Lacey, Karen Powell Sears, Ishtar O. Govia, Ivy Forsythe-Brown, Niki Matusko, James S. Jackson

**Affiliations:** 1 Program for Research on Black Americans, Institute for Social Research, University of Michigan, 5062 ISR Building 426 Thompson St., Ann Arbor, MI 48106, USA; E-Mails: ishtargovia@gmail.com (I.O.G.); snk@umich.edu (N.M.); jamessj@umich.edu (J.S.J.); 2 Department of Sociology and Anthropology, Denison University, 100 West College Street, Granville, OH 43023, USA; E-Mail: searsk@denison.edu; 3 Department of Sociology, Psychology and Social Work, The University of the West Indies, Mona, Kingston 7, Jamaica; 4 Department of Behavioral Sciences, University of Michigan-Dearborn, 4901 Evergreen Rd, Dearborn, MI 48128, USA; E-Mail: ivyfb@umich.edu

**Keywords:** substance use, mental and physical health, culture, ethnicity, migration

## Abstract

This study compares the health conditions of domestic Caribbeans with those living in the United States to explore how national context and migration experiences might influence substance use (*i.e.*, alcohol or drug) and other mental and physical health conditions. The study is based upon probability samples of non-institutionalized Caribbeans living in the United States (1621), Jamaica (1216) and Guyana (2068) 18 years of age and over. Employing descriptive statistics and multivariate analytic procedures, the results revealed that substance use and other physical health conditions and major depressive disorder and mania vary by national context, with higher rates among Caribbeans living in the United States. Context and generation status influenced health outcomes. Among first generation black Caribbeans, residing in the United States for a longer length of time is linked to poorer health outcomes. There were different socio-demographic correlates of health among at-home and abroad Caribbeans. The results of this study support the need for additional research to explain how national context, migratory experiences and generation status contribute to understanding substance use and mental disorders and physical health outcomes among Caribbean first generation and descendants within the United States, compared to those remaining in the Caribbean region.

## 1. Introduction

The Caribbean population in the United States is rapidly expanding. An estimated one in two Black immigrants in the United States is of Caribbean origin [1]. Many of these immigrants are from Jamaica and Guyana, two of the largest sources of Caribbean-US migration. In search of a better quality of life, Caribbean immigrants might encounter post-migration social and structural barriers that over time can negatively affect their health. The effects of migration may serve to gradually erode the health advantages that these Caribbean migrant groups have been thought to enjoy relative to US-born populations.

The belief that individuals living in the Caribbean have better health than those born in the United States has been attributed to a number of factors, including a generally more relaxed and healthier lifestyle. There has been, however, little empirical evidence to support these assumptions [2]. Much of the knowledge gained about the health status of Caribbeans has been based on samples collected in one geographic location. No study, to our knowledge, has addressed the health status of Caribbean descendants within and outside Caribbean nations. Research in this area is necessary to help address claims that social context and processes of migration might contribute to deteriorating health. This study examines the substance use and the mental and physical health of at-home and abroad black Caribbeans.

### 1.1. Background

The migration literature largely reports that immigrant groups have more favorable health than their US-born counterparts [3,4,5,6,7,8]. A growing number of studies suggest that increased time and exposure to the US lifestyle is correlated with deteriorating health [3,5,6,9,10,11]. This notion has largely been supported among various immigrant groups within the United States, including Hispanics, Asian Americans and Afro-Caribbeans [5,6,9,10,12]. The advantaged health status of immigrants has been attributed to the healthy immigrant effect; the notion that differences in socio-cultural aspects of diet, physical activity, nutrition and the use of tobacco and alcohol that exist between the migrant’s place of origin and the host country contribute positively to immigrant health [13].

The process by which individuals from the Caribbean region are selected for entrance to the United States may also contribute to explaining their advantaged health standing over non-immigrants. Prior to migration to the United States, individuals generally undergo medical screening procedures that detect for illnesses and diseases (e.g., communicable diseases), which if serious enough, can disqualify them from entry into the country. Through this process, only those with generally good health are selected for entrance [14].

While gaining entrance to the United States can present its own set of challenges for some immigrant groups, the migratory experience can indirectly and directly shape the health of individuals. Increased potential for mental health stressors may exist with changes in geographic locations [15]. Despite the possibility for new opportunities in the host country, migration is typically associated with loss of social and personal support networks. While some immigrants are optimistic about the prospect of new opportunities in host countries and possibilities of re-unification with family members, changes in environment can cause culture shock and isolation. The unfamiliar and unpredictable nature of new personal and institutional relationships in the host country can produce anxiety, stress and other negative health risks [3]. Additionally, immigrants may experience acculturative stress, a type of cultural dissonance as they are faced with a new way of life that counters existing beliefs and values, potentially causing stress and altering psychological states, that may encourage the use of substances and contributing to other negative health conditions [3,16,17].

Negative mental and physical conditions may be further heightened due to barriers encountered during the transition to their new homeland. For example, migrants may have difficulties finding employment because they lack the requisite skills necessary to compete effectively in the new labor market. Even with appropriate credentials, their qualifications might not be viewed as comparable to those required within the host country, causing denial of employment opportunities. Denial of opportunities can cause many new immigrants to accept menial and low paying jobs, which may not match their occupational training, experience and/or expectations, further causing distress and frustration [18,19]. Limited financial resources and opportunities that many immigrants face after migration may subject them to substandard housing in less desirable neighborhoods with high criminal activity and environmental contaminants. The physical and social dangers of these environments can encourage substance use and lead to other poor health conditions, such as depression, anxiety, hypertension and cancer [14,20,21].

The possibility for poor health conditions among migrants may be further exacerbated with unfamiliar discriminatory practices not present in their homelands [3,22,23,24]. Since some recent immigrants, especially from the English-speaking Caribbean, tend to come from more privileged backgrounds in their home countries [25,26], these challenges may be more daunting for health and well-being due to difficulties in adjusting to their new living arrangements [27]. This disappointment can affect individuals’ psychological dispositions and may contribute to the use of substances to cope with their life circumstances.

Despite challenges in host countries, some migrants may have already been exposed to circumstances in their homeland that negatively affect their health. Certain migrant populations may have lower quality of life and a higher risk for infectious diseases, cancer, diabetes and heart disease [13]. In high sending countries, such as Guyana and Jamaica, widespread unemployment and poverty remain pressing issues, and have been associated with notable rates of depression and other negative physical health conditions [28,29]. Within these countries, affordable health care is limited and many citizens lack the financial means to obtain it when available. The recent economic downturn has added to the dire economic situation, creating rippling effects on institutions responsible for providing care, and promoting healthier habits among its citizens [30].

Natural catastrophes and political unrest in certain Caribbean regions can be traumatic and negative to the health of individuals. For example, psychological distress resulting from natural disasters not only increases the risk of developing mental disorders such as depression and mania, but also can exacerbate pre-existing conditions [31,32]. High rates of crime and political violence within the Caribbean region have also created fear, while intensifying the risk for substance use, anxiety, depression and post-traumatic stress disorder among individuals [33,34,35].

Industrialization and technological advancements have also been regarded as possible contributors to risky behaviors and poor health among citizens in the Caribbean. While these advancements have modernized the Caribbean region, they have also altered traditional ways of life, creating dependency on mechanized transportation and encouraging sedentary lifestyles [22]. This has given rise to more pollution and higher rates of inactivity and obesity, which have been associated with several prevalent health conditions, including diabetes [33,36].

For these and other reasons, it may be difficult to empirically substantiate claims that descendants within Caribbean regions have better health relative to migrants to the United States, and that social context and processes related to migration contribute to deteriorating health. Complicating these assumptions is the lack of cross-national population studies on the health of Caribbean persons within and outside the Caribbean regions. This study examined the health status of Caribbean persons across three geographic locations: Guyana, Jamaica and the United States. The association of socio-demographic factors, social context and generation status on the health disposition of Caribbean descendants in the United States and Caribbean regions was also examined.

### 1.2. Conceptual Frameworks

This study is informed by the various frameworks that have provided explanations for the health outcomes associated with migration. Foundational ideas from social learning, acculturation, and structural frameworks have value for understanding these potential health outcomes. Social learning theory describes the process by which behaviors are learned and changed [37,38,39]. According to this framework, learning occurs through modeling, vicarious learning and reinforcement. When learning is perceived to be advantageous as a result of social and/or personal rewards, it is repeated [40]. This perspective can improve understanding of key elements of acculturation, the processes by which adapting elements of American culture can have negative implications for immigrant health.

The acculturation hypothesis describes the process by which immigrant groups learn cultural knowledge, behavior and attitudes. Cultural information influencing health behavior and characteristics may also emerge though this learning process. Through the processes of acculturation, immigrant groups subvert elements of their native culture and adopt behaviors and characteristics of the host culture. Various contextual factors may shape the speed and content of acculturation, with persons differing in rate, degree and content of native and host cultural, personal and behavioral attributes that are retained and lost. The process of acculturation is often gradual and tends to progress generationally. Those who have spent more time in the host culture have greater exposure to the host country’s cultural practices and norms and are more likely to adapt various cultural elements as a result of both direct and indirect influences [41,42,43,44]. With regard to the health related practices and attitudes of immigrant groups, the acculturation model posits that over time immigrants’ health related attitudes and behaviors will resemble the modal attitudes and behaviors of natives in the host country.

The learning of attitudes that influence health behaviors may occur both directly or indirectly. Persons may experience greater direct exposure to unhealthy practices, such as poor diets or sedentary lifestyles, or the use of drugs and alcohol as a means of coping. Persons may also be indirectly influenced by being in a cultural environment that promotes the use of unhealthy food, drugs and alcohol as normative social practice, as well as a means for managing difficulties. For instance, various studies have reported increased rates of alcohol and tobacco use among immigrant groups who have had longer periods of exposure in North American countries [45,46].

Finally, structural theory proposes that social structural conditions, such as poverty, lower levels of education, high unemployment and racial discrimination in host countries, are sources of stress that are unevenly distributed in the social structure. These structural factors in host nations may adversely affect the physical and mental health of immigrants, particularly those considered racial and ethnic minorities in the host countries [38,47].

### 1.3. Statement of Hypotheses

In line with these frameworks, we expect that poorer mental and physical conditions will be higher among immigrants who are more highly acculturated and have spent longer periods of time in the United States. Risk for poor mental and physical health is further expected to be higher among socially and economically disadvantaged individuals.

## 2. Methods

Three datasets were analyzed independently. The National Survey of American Life (NSAL) was collected in the United States, and the Jamaica and Guyana cross-section data were collected in the respective countries:

*United States*. The National Survey of American Life (NSAL) conducted between 2001 and 2003 is presently the most comprehensive and detailed study on the physical and mental disorders of individuals of African descent living in the United States, and the first national study of US Caribbean Blacks. The NSAL used a multi-stage area sample design that combines a core national area probability sample with a special supplemental sample of households in areas of higher Caribbean Black residential density to generate a national sample of Caribbeans living in the United States. High population density areas that contained more than 80% of the Caribbean descent targeted included New York, New Jersey, Washington DC, Florida, Connecticut, and Massachusetts. To qualify for the study individuals self-reported Caribbean roots by nature of migration or relationship with parents or grandparents that were born in the Caribbean, or who were from the list of Caribbean countries presented by the interviewers [48,49]. In-person interviews and questionnaires were used to gather information on participants who met qualifying criteria. A total of 6082 adults over the age of 18 completed the survey in English: 891 non-Hispanic Whites; 3570 African Americans; and 1621 first and subsequent generation individuals of English, Spanish, French and Dutch Caribbean descent. The response rate for the Caribbean population was 77.7%.

*Jamaica*. The sample in Jamaica consists of randomly selected adults living in the Kingston metropolitan area. Samples were generated from the 2002 census tract urban regions of Kingston and St. Andrew, and Portmore [50]. Interviews with study participants were conducted in August and completed in December of 2005. A total of 1216 interviews were collected, with an overall response rate of 76%. The sample consisted of roughly 97.4% Blacks, 1.3% Asians, and 1.4% who were classified as “Other”.

*Guyana*. In Guyana the sample consists of randomly selected adults living in greater Georgetown (urban) and other rural and suburban areas. The samples collected were based on the 2001 census regions [51]. Questionnaires were administered to Guyanese participants between July and December 2005. A total of 2068 participants completed questionnaires with an overall response rate of 82%. There were 55.2% Blacks, 34.7% East Indian, and 10.1% Mixed/other races in the sample.

### 2.1. Substance Use and Mental Disorders and Physical Health Outcome Measures

*Substance Use and Mental Disorders*. This study addresses the lifetime occurrence of Diagnostic and Statistical Manual of Mental Disorders, Fourth Edition (DSM-IV) mental health conditions including alcohol abuse, drug abuse, major depressive disorder, and mania. These disorders were assessed using a slightly modified version of the World Health Organization’s World Mental Health Composite International Diagnostic Interview (WMH CIDI). The phrase “substance abuse” refers to the presence of either alcohol or drug abuse, or both. The criteria for substance abuse—drug abuse or alcohol abuse—includes Criteria A (a maladaptive pattern of use leading to clinically significant impairment or distress), and B (the symptoms have never met the criteria for dependence for that substance).

The algorithm for Major Depressive Disorder is the same as that for Major Depressive Episode: MDE Criteria C, the presence or absence of a manic, mixed or hypomanic episode is not considered. The individual must have had Criteria A, at least five depressive symptoms, representing a change in functioning, during the same 2 week period, and at least one of the symptoms is depressed mood or loss of interest or pleasure; C, the symptoms cause clinically significant distress or cause impairment in functioning; and D, the symptoms are not due to the physiological effects of a substance or general medical condition. Episodes due to bereavement were not excluded.

Mania is defined as meeting Criteria A, a distinct period of abnormally and persistently elevated, expansive or irritable mood lasting at least 1 week; Criteria B, three or more symptoms have persisted and Criteria D, the mood disturbance is sufficiently severe to cause marked impairment in functioning or in usual social activities or relationships and E, the symptoms are not due to the direct physiological effects of a substance or general medical condition.

*Physical Health*. Five indicators of physical health were examined. Participants across samples were asked (yes/no) to indicate whether they had ever been diagnosed by a doctor for hypertension or “high blood pressure”, diabetes or “sugar”, and arthritis or “rheumatism.” They were further asked to rate their dental and physical health on a Likert-like scale such as excellent, very good, good, fair and poor self-rated health. In this study, we focused on a dichotomized response category of fair/poor *vs.* very good/good/excellent for both self-rated indicators.

### 2.2. Social and Economic Correlates

*Socio-demographic Variables*. The socio-demographic predictors of health included: age, gender (male, female), marital status (e.g., never married; married; partnered; separated, divorced or widowed), education level (e.g., primary/some high school; high school graduate; college, vocational or technical), employment status (e.g., employed; not employed; not in labor force) and, income in quintiles (e.g., bottom; second; middle; fourth; and highest).

*Ancestry*. Exposure of Caribbean descendants to the United States context was assessed by examining their ancestry. *First generations* (under and over 20 years of residence) were those who had directly migrated to the United States from Caribbean regions. For the purpose of this study we coded the variable into those who have lived in the US for fewer than 20 years and those who have lived in the US for more than 20 years, as prior research on substance use and mental health among Caribbeans using the NSAL suggests that this is a key transition point for these outcomes, although we are aware of other time-in-country breaks that may also be relevant [6].

*Second generation* participants were born in the United States to at least one Caribbean immigrant parent. *Third generations* consisted of US born blacks whose parents were born in the United States and had Caribbean born grandparents. In this study, we did not directly assess acculturation experiences of the participants in the United States.

### 2.3. Analytic Strategy

Descriptive statistics were used to examine lifetime prevalence rates of substance use, mental and physical health across the three main geographic locations. We then employed multivariate logistic regression analytic procedures to calculate odds ratio and confidence intervals while adjusting for other factors within countries. Significance was set at the conventional 0.05 alpha level. Due to the underlying complex sample design of the US sample, standard errors were corrected for weighting; clustering and stratification adjustments were made for complex sample designs, and differential non-response. Post-stratification weights were applied based on national population distributions for gender and age subgroups in Guyana and Jamaica.

### 2.4. Sample Characteristics

As shown in Table 1, the samples across countries revealed that Jamaican participants were younger (M_age_ = 37.2 years) than members of other cohorts. Females comprised the majority across samples, with a higher percentage in Jamaica (70%). More Guyanese (34%) and US Caribbeans (37.6%) were married compared to a majority of Jamaicans who were never married (58.5%). US Caribbean participants generally had higher education levels (e.g., college, vocational, or technical) (49.1%) than participants in Caribbean regions. While participants across samples were generally employed, a higher percentage was found among US Caribbeans (75.2%). Similarly, a higher percentage of US Caribbeans (30%) were represented in the highest income quintile category than participants in Caribbean regions. Also, more Guyanese (59.4%) owned homes compared to Jamaicans (47.2%) and US Caribbeans (44.9%), who generally rented.

**Table 1:** Sample characteristics.

Characteristics	Guyana (%)	Jamaica (%)	US Caribbeans (%)
Mean Age	40.5	37.2	40.3
Gender			
Male	48.2	29.8	50.9
Female	51.8	70.2	49.1
Marital Status			
Never married	31.2	58.5	30.9
Married	34.2	19.5	37.6
Partnered	16.0	13.6	12.6
Sep-Div-Widow	18.6	8.5	18.9
Education			
Primary/Some HS	54.0	26.1	21.2
High School Grad	29.7	51.8	29.7
College-Voc-Tech	16.3	22.1	49.1
Employment Status			
Employed	53.7	45.2	75.2
Unemployed	10.8	29.6	8.8
Not in Labor Force	35.5	25.2	16.0
Equivalized Income			
Bottom Quintile	14.0	19.7	14.0
Second Quintile	30.0	24.1	14.6
Middle Quintile	23.4	1.5	20.8
Fourth Quintile	22.4	43.6	19.1
Highest Quintile	10.2	11.1	31.6
Home Ownership			
Does Not Own	40.6	52.8	55.1
Own	59.4	47.2	44.9
*[N]*	*2068*	*1218*	*1623*

Note: All estimates are weighted to be nationally representative of the given population groups in the continental U.S. and respective two Caribbean countries.

## 3. Results and Discussion

### 3.1. Prevalence Rates Substance Use, Mental and Physical Health

**Figure 1:**
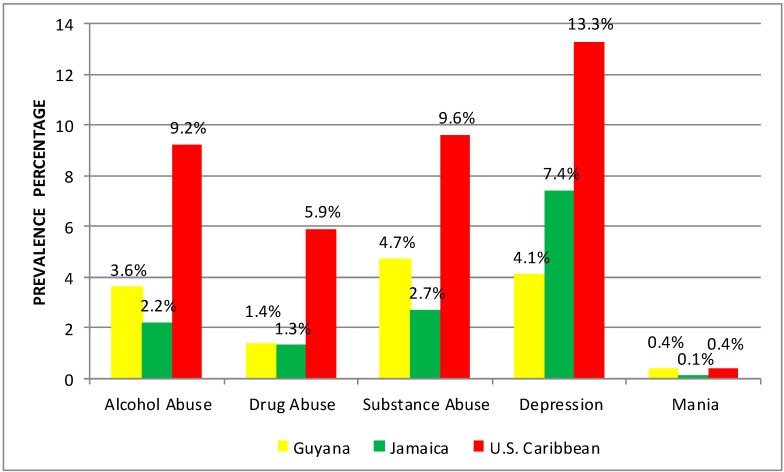
Lifetime prevalence of substance use and mental disorders by country.

Figure 1 shows differences in rates of substance use and mental disorders across geographic regions (see Figure 1). Across conditions, rates were higher among Caribbean descendants in the United States for alcohol abuse (9.2%), drug abuse (5.9%), substance abuse (9.6%), and depression (13.3%). Comparable rates were found for mania between US Caribbeans and Guyanese (0.4%* vs.* 0.4%). However, within Caribbean regions mental disorder rates were generally higher for Guyanese than Jamaicans.

**Figure 2:**
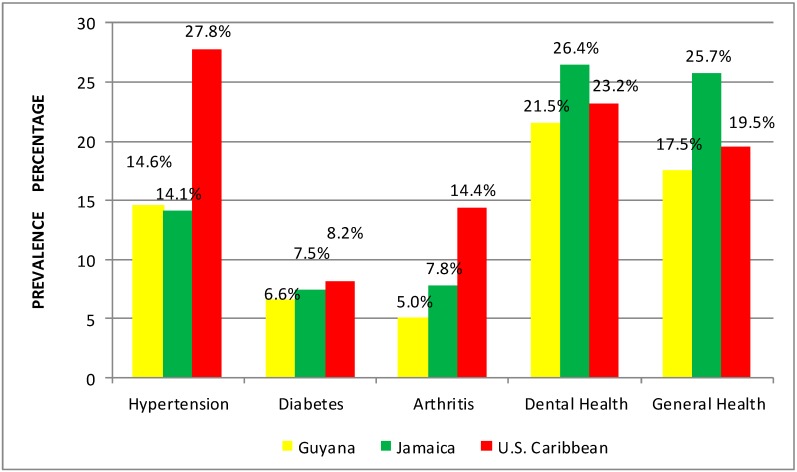
Lifetime prevalence of physical health by country.

Figure 2 also reveals that Caribbean descendants within the United States have higher lifetime rates of hypertension (27.8%), diabetes (8.2%) and arthritis (14.4%). Jamaicans on the contrary had greater prevalence of fair to poor self-rated dental (26.4%) and physical health (25.7%) than other groups. They also generally had poorer physical health than their Guyanese Caribbean counterparts.

### 3.2. Multivariate Results

#### 3.2.1. Substance Use and Mental Health Disorders

Table 2 illustrates that gender was predictive of alcohol abuse across countries. Females in Guyana (Adjusted Odds Ratio (AOR) = 0.15, *p* < 0.001), Jamaica (AOR = 0.11, *p* < 0.001), and the United States (AOR = 0.08, *p* < 0.001) had lower odds of alcohol abuse compared to their male counterparts. 

**Table 2:** Factors associated with lifetime prevalence of alcohol abuse by country.

Characteristics	Guyana	Jamaica	US Caribbeans
	AOR (CI)	AOR (CI)	AOR (CI)
Age	1.01 (0.99–1.03)	1.04 (1.01–1.07) ^*^	1.00 (0.95–1.05)
Gender			
Male	1	1	1
Female	0.15 (0.08–0.31) ^***^	0.11 (0.04–0.29) ^***^	0.08 (0.03–0.19) ^***^
Marital Status			
Never Married	1	1	1
Married	0.92 (0.48–1.74)	0.77 (0.20–3.04)	2.23 (0.33–14.96)
Partnered	1.22 (0.60–2.50)	4.47 (1.44–13.87) ^**^	4.29 (0.56–32.99)
Sep-Div-Widow	1.16 (0.53–2.53)	1.49 (0.39–5.77)	1.76 (0.17–18.23)
Education			
Primary/Some HS	1	1	1
High School Grad	0.89 (0.48–1.67)	0.34 (0.09–1.31)	0.25 (0.04–1.37)
College-Voc-Tech	1.00 (0.51–1.97)	0.08 (0.01–0.82)	0.76 (0.27–2.15)
Employment Status			
Employed	1	1	1
Unemployed	1.58 (0.76–3.30)	1.72 (0.52–5.65)	1.12 (0.49–2.57)
Not in Labor Force	0.43 (0.20–0.914)^ *^	0.39 (0.11–1.38)	1.10 (0.25–4.96)
Equivalized Income			
Bottom Quintile	1	1	1
Second Quintile	0.68 (0.32–1.45)	0.59 (0.18–1.93)	0.34 (0.11–1.03)
Middle Quintile	0.67 (0.30–1.49)	n/a	0.43 (0.07–2.73)
Fourth Quintile	1.36 (0.64–2.86)	2.32 (0.47–11.44)	0.92 (0.31–2.71)
Highest Quintile	0.35 (0.10–1.27)	1.51 (0.17–13.09)	0.26 (0.07–0.99) ^*^
Ancestry			
<20 years	--	--	1
>20 years	--	--	2.59 (0.52–12.87)
Second Generation	--	--	20.35 (4.33–95.61) ^***^
Third Generation	--	--	10.28 (1.94–54.49) ^**^

Notes: “AOR” refers to Adjusted Odds Ratio and “n/a” indicates there was insufficient data. ^*^ *p* < 0.05; ^**^ *p* < 0.01; ^***^ *p* < 0.001.

Lower odds of alcohol abuse was also found among Guyanese not in the labor force (AOR = 0.43, *p* < 0.01) as well as US Caribbeans within the highest income quintile (AOR = 0.26, *p* < 0.05). However, the odds for alcohol abuse significantly increased with age among Jamaican respondents (AOR = 1.04, *p* < 0.05). This increase was also observed for second (AOR = 20.35, *p* < 0.001) and third (AOR = 10.28, *p* < 0.05) generation Caribbeans, compared to first generation participants who had migrated to the United States for less than 20 years. The likelihood for alcohol abuse was also found among married Jamaicans (AOR = 4.47, *p* < 0.01).

As shown in Table 3, gender was also predictive of drug abuse across contexts. Compared to males, the odds of drug abuse was reduced among Guyanese (AOR = 0.09, *p* < 0.001), Jamaican (AOR = 0.28, *p* < 0.05) and US Caribbean (AOR = 0.14, *p* < 0.001) female participants. 

**Table 3:** Factors associated with lifetime prevalence of drug abuse by country.

Characteristics	Guyana	Jamaica	US Caribbeans
	AOR (CI)	AOR (CI)	AOR (CI)
Age	0.98 (0.95–1.02)	1.01 (0.96–1.05)	0.99 (0.97–1.02)
Gender			
Male	1	1	1
Female	0.09 (0.02–0.36) ^***^	0.28 (0.09–0.88) ^*^	0.14 (0.07–2.74) ^***^
Marital Status			
Never Married	1	1	1
Married	0.11 (0.02–0.72) ^*^	n/a	0.36 (0.08–1.54)
Partnered	0.66 (0.21–2.08)	1.42 (0.25–7.90)	2.84 (0.51–15.69)
Sep-Div-Widow	2.09 (0.75–5.86)	2.77 (0.55–13.81)	0.54 (0.10–2.90)
Education			
Primary/Some HS	1	1	1
High School Grad	1.08 (0.44–2.67)	0.28 (0.04–1.93)	0.16 (0.04–0.75) ^*^
College-Voc-Tech	0.25 (0.06–1.11)	0.12 (0.01–2.07)	0.69 (0.20–2.37)
Employment Status			
Employed	1	1	1
Unemployed	0.98 (0.25–3.82)	1.45 (0.36–5.89)	1.38 (0.48–3.97)
Not in Labor Force	0.12 (0.01–1.01) ^*^	0.24 (0.04–1.40)	1.33 (0.24–7.38)
Equivalized Income			
Bottom Quintile	1	1	1
Second Quintile	0.59 (0.11–3.20)	0.35 (0.06–1.97)	0.45 (0.12–1.64)
Middle Quintile	2.21 (0.54–9.00)	n/a	0.59 (0.12–2.93)
Fourth Quintile	1.83 (0.44–7.69)	1.62 (0.19–13.68)	0.67 (0.22–2.02)
Highest Quintile	0.86 (0.10–7.24)	n/a	0.11 (0.02–0.68) ^*^
Ancestry			
<20 years	--	--	1
>20 years	--	--	15.08 (1.94–117.04) ^**^
Second Generation	--	--	35.56 (8.92–141.79) ^***^
Third Generation	--	--	15.50 (2.59–92.85) ^**^

Notes: “AOR” refers to Adjusted Odds Ratio and “n/a” indicates there was insufficient data. ^*^ *p* < 0.05; ^**^ *p* < 0.01; ^***^ *p* < 0.001.

The lower odds in drug abuse was further observed among US Caribbean participants with a high school education (AOR = 0.16, *p* < 0.05) and those within the highest income category (AOR = 0.11, *p* < 0.05). This lower odds in drug use was further evident for Guyanese participants who were married (AOR = 0.11, *p* < 0.05) and not in the labor force (AOR = 0.12, *p* = 0.051). By contrast, the odds for drug abuse significantly increased among first generation Caribbeans who had resided in the United States for over 20 years (AOR = 15.08, *p* < 0.01), and among second (AOR = 35.56, *p* < 0.001) and third (AOR = 15.50, *p* < 0.01) generations.

As indicated in Table 4, there was an association between gender and substance abuse. Again, there were lower odds for substance abuse among Guyanese (AOR = 0.12, *p* < 0.001), Jamaican (AOR = 0.15, *p* < 0.001) and US Caribbean females (AOR = 0.10, *p* < 0.001). This reduction in substance abuse was also found among Guyanese that were not in the labor force (AOR = 0.39, *p* < 0.01). 

**Table 4:** Factors associated with lifetime prevalence of substance abuse by country.

Characteristics	Guyana	Jamaica	US Caribbeans
	AOR (CI)	AOR (CI)	AOR (CI)
Age	1.00 (0.99–1.02)	1.03 (1.00–1.06) ^*^	1.00 (0.95–1.05)
Gender			
Male	1	1	1
Female	0.12 (0.06–0.23) ^***^	0.15 (0.07–0.36) ^***^	0.10 (0.05–0.20) ^***^
Marital Status			
Never Married	1	1	1
Married	0.66 (0.37–1.20)	0.67 (0.18–2.50)	1.92 (0.32–11.52)
Partnered	0.99 (0.53–1.88)	3.28 (1.14–9.47) ^*^	4.13 (0.62–27.67)
Sep-Div-Widow	1.54 (0.80–2.96)	2.03 (0.61–6.78)	1.65 (0.18–14.99)
Education			
Primary/Some HS	1	1	1
High School Grad	0.87 (0.51–1.49)	0.52 (0.16–1.74)	0.27 (0.06–1.28)
College-Voc-Tech	0.63 (0.33–1.19)	0.18 (0.03–1.16)	0.69 (0.26–1.82)
Employment Status			
Employed	1	1	1
Unemployed	1.48 (0.75–2.93)	1.73 (0.60–5.03)	0.99 (0.43–2.28)
Not in Labor Force	0.39 (0.19–0.78) ^**^	0.35 (0.11–1.13)	1.19 (0.28–5.15)
Equivalized Income			
Bottom Quintile	1	1	1
Second Quintile	0.63 (0.31–1.32)	0.52 (0.17–1.60)	0.42 (0.14–1.26)
Middle Quintile	0.99 (0.48–2.00)	n/a	0.45 (0.08–2.65)
Fourth Quintile	1.64 (0.82–3.29)	1.51 (0.37–6.28)	0.94 (0.32–2.70)
Highest Quintile	0.50 (0.16–1.51)	0.71 (0.10–5.15)	0.32 (0.09–1.15)
Ancestry			
<20 years	--	--	1
>20 years	--	--	2.22 (0.49–10.14)
Second Generation	--	--	16.29 (4.13–64.26) ^***^
Third Generation	--	--	8.55 (1.81–40.25) ^**^

Notes: “AOR” refers to Adjusted Odds Ratio and “n/a” indicates there was insufficient data. ^*^ *p* < 0.05; ^**^ *p* < 0.01; ^***^ *p* < 0.001.

In contrast, a significant increase in odds for substance abuse was found for both second (AOR = 16.29, *p* < 0.001) and third (AOR = 8.55, *p* < 0.01) generation US Caribbeans. Partnered (AOR = 3.28, *p* < 0.05) and aging (AOR = 1.03,* p* < 0.05) Jamaican participants also had higher odds to abuse substances. 

As shown in Table 5, there were various predictors of major depressive disorder (MDD) across nations. The odds (AOR = 0.98,* p* < 0.05) for depression was reduced for older participants in Jamaica. Similarly, married (AOR = 0.43, *p* < 0.05) and unemployed US Caribbeans (AOR = 0.61, *p* < 0.05) had lower odds of depression. Conversely, the odds for depression increased among third generation (AOR = 4.05, *p* < 0.01) US Caribbeans. Analysis on mania was not conducted due to low prevalence rates of the disorder.

**Table 5:** Factors associated with lifetime prevalence of major depressive disorder by country.

Characteristics	Guyana	Jamaica	US Caribbeans
	AOR (CI)	AOR (CI)	AOR (CI)
Age	1.00 (0.979–1.02)	0.98 (0.96–0.995) ^*^	0.99 (0.97–1.02)
Gender			
Male	1	1	1
Female	1.44 (0.88–2.38)	1.49 (0.86–2.59)	1.02 (0.53–1.97)
Marital Status			
Never Married	1	1	1
Married	0.55 (0.29–1.04)	0.85 (0.42–1.72)	0.43 (0.22–0.84) ^*^
Partnered	0.81 (0.41–1.61)	0.75 (0.36–1.59)	0.92 (0.39–2.20)
Sep-Div-Widow	1.01 (0.49–2.05)	1.86 (0.84–4.14)	0.90 (0.37–2.19)
Education			
Primary/Some HS	1	1	1
High School Grad	1.25 (0.71–2.21)	0.84 (0.39–1.79)	0.61 (0.16–2.33)
College-Voc-Tech	0.56 (0.24–1.32)	0.90 (0.84–2.14)	1.42 (0.45–4.47)
Employment Status			
Employed	1	1	1
Unemployed	0.83 (0.39–1.76)	1.31 (0.65–2.63)	0.61 (0.38–0.98) ^*^
Not in Labor Force	0.74 (0.41–1.31)	0.62 (0.32–1.21)	0.86 (0.40–1.86)
Equivalized Income			
Bottom Quintile	1	1	1
Second Quintile	0.59 (0.30–1.15)	0.90 (0.43–1.88)	1.39 (0.34–5.77)
Middle Quintile	0.53 (0.26–1.09)	2.08 (0.41–10.50)	1.40 (0.25–7.90)
Fourth Quintile	0.56 (0.26–1.18)	0.92 (0.37–2.28)	1.33 (0.36–4.95)
Highest Quintile	0.53 (0.20–1.38)	0.91 (0.34–2.40)	0.64 (0.15–2.75)
Ancestry			
<20 years	--	--	1
>20 years	--	--	1.17 (0.53–2.59)
Second Generation	--	--	1.67 (0.46–6.09)
Third Generation	--	--	4.05 (1.38–11.84) ^*^

Notes: “AOR” refers to Adjusted Odds Ratio and “n/a” indicates there was insufficient data. ^*^ *p* < 0.05; ^**^ *p* < 0.01; ^***^ *p* < 0.001.

#### 3.2.2. Physical Health

Table 6 illustrates that in Guyana (AOR = 1.09, *p* < 0.001), Jamaica (AOR = 1.06, *p* < 0.001) and among US Caribbean respondents (AOR = 1.07, *p* < 0.001) the odds of hypertension increased with age. Hypertension also increased among female Guyanese (AOR = 1.96, *p* < 0.01) and Jamaican (AOR = 3.67, *p* < 0.001) participants compared to men within the respective populations. Similar increases were found among US Caribbeans within the highest income quintile category (AOR = 2.87, *p* < 0.001) and among third generation participants (AOR = 3.02, *p* < 0.01). Increased odds were also observed among married (AOR = 2.06*, p* < 0.01) and partnered (AOR = 2.01, *p* < 0.05) Guyanese participants. There, however, were reduced odds for hypertension among college-educated participants within this population (AOR = 0.52, *p* < 0.05).

**Table 6:** Factors associated with lifetime prevalence of hypertension by country.

Characteristics	Guyana	Jamaica	US Caribbeans
	AOR (CI)	AOR (CI)	AOR (CI)
Age	1.09 (1.08–1.11) ^***^	1.06 (1.04–1.08) ^***^	1.07 (1.04–1.10) ^***^
Gender			
Male	1	1	1
Female	1.96 (1.39–2.78) ^***^	3.67 (2.14–6.28) ^***^	1.15 (0.64–2.06)
Marital Status			
Never Married	1	1	1
Married	2.06 (1.26–3.35) ^**^	1.36 (0.81–2.29)	0.93 (0.45–1.94)
Partnered	2.01 (1.11–3.65) ^*^	1.07 (0.52–2.22)	0.75 (0.27–2.12)
Sep-Div-Widow	0.99 (0.57–1.70)	1.10 (0.56–2.19)	0.50 (0.12–2.21)
Education			
Primary/Some HS	1	1	1
High School Grad	0.83 (0.54–1.29)	1.04 (0.51–2.11)	0.52 (0.17–1.57)
College-Voc-Tech	0.52 (0.30–0.90) *	0.743 (0.31–1.79)	0.60 (0.22–1.70)
Employment Status			
Employed	1	1	1
Unemployed	0.84 (0.47–1.48)	0.76 (0.39–1.46)	1.14 (0.59–2.22)
Not in Labor Force	1.01 (0.68–1.50)	0.96 (0.56–1.64)	1.54 (0.82–2.90)
Equivalized Income			
Bottom Quintile	1	1	1
Second Quintile	0.81 (0.52–1.26)	1.30 (0.72–2.34)	1.60 (0.64–4.01)
Middle Quintile	1.09 (0.64–1.85)	1.01 (0.11–9.17)	1.78 (0.49–6.38)
Fourth Quintile	0.73 (0.41–1.31)	1.04 (0.46–2.36)	1.42 (0.50–4.00)
Highest Quintile	0.72 (0.36–1.43)	1.07 (0.37–3.10)	2.87 (1.80–4.59) ^***^
Ancestry			
<20 years	--	--	1
>20 years	--	--	1.19 (0.49–2.92)
Second Generation	--	--	1.78 (0.55–5.78)
Third Generation	--	--	3.02 (1.44–6.34) ^**^

Notes: “AOR” refers to Adjusted Odds Ratio and “n/a” indicates there was insufficient data. ^*^ *p* < 0.05; ^**^ *p* < 0.01; ^***^ *p* < 0.001.

Table 7 again shows that age was associated with being diagnosed with diabetes among participants across countries with Guyanese (AOR = 1.05, *p* < 0.001), Jamaican (AOR = 1.07, *p* < 0.001) and US Caribbean (AOR = 1.09,* p* < 0.001) participants having greater odds for diabetes as they aged. US Caribbean (AOR = 3.24, *p* < 0.01) and Jamaican (AOR = 2.36, *p* < 0.01) women also had greater odds for diabetes. In contrast to these findings, there was reduced odds for diabetes among high school (AOR = 0.48, *p* < 0.05) and college educated (AOR = 0.23, *p* < 0.001) Guyanese participants. Just the same, lower odds of diabetes was found among unemployed Guyanese participants (AOR = 0.40, *p* < 0.05).

**Table 7:** Factors associated with lifetime prevalence of diabetes by country.

Characteristics	Guyana	Jamaica	US Caribbeans
	AOR (CI)	AOR (CI)	AOR (CI)
Age	1.05 (1.03–1.07) ^***^	1.07 (1.05–1.09) ^***^	1.09 (1.06–1.12) ^***^
Gender			
Male	1	1	1
Female	1.47 (0.94–2.29)	2.36 (1.21–4.57) ^**^	3.24 (1.63–6.46) ^**^
Marital Status			
Never Married	1	1	1
Married	1.60 (0.92–2.79)	1.17 (0.59–2.32)	1.69 (0.66–4.32)
Partnered	0.46 (0.18–1.16)	0.58 (0.18–1.80)	1.16 (0.38–3.52)
Sep-Div-Widow	0.78 (0.40–1.54)	1.02 (0.45–2.32)	0.82 (0.34–1.94)
Education			
Primary/Some HS	1	1	1
High School Grad	0.48 (0.26–0.88) ^*^	0.51 (0.21–1.25)	0.50 (0.23–1.11)
College-Voc-Tech	0.23 (0.08–0.54) ^***^	0.72 (0.25–2.09)	0.40 (0.14–1.13)
Employment Status			
Employed	1	1	1
Unemployed	0.40 (0.17–0.913) ^*^	1.49 (0.68–3.22)	2.70 (0.97–7.50)
Not in Labor Force	0.70 (0.42–1.18)	0.46 (0.20–1.05)	0.65 (0.23–1.83)
Equivalized Income			
Bottom Quintile	1	1	1
Second Quintile	1.07 (0.60–1.90)	1.49 (0.75–2.97)	0.47 (0.18–1.24)
Middle Quintile	0.66 (0.31–1.42)	n/a	0.71 (0.21–2.45)
Fourth Quintile	0.72 (0.33–1.54)	0.94 (0.30–2.91)	1.67 (0.71–3.93)
Highest Quintile	1.26 (0.56–2.87)	1.42 (0.41–4.93)	1.68 (0.69–4.10)
Ancestry			
<20 years	--	--	1
>20 years	--	--	0.49 (0.22–1.10)
Second Generation	--	--	0.54 (0.18–1.63)
Third Generation	--	--	1.80 (0.44–7.46)

Notes: “AOR” refers to Adjusted Odds Ratio and “n/a” indicates there was insufficient data. ^*^ *p* < 0.05; ^**^ *p* < 0.01; ^***^ *p* < 0.001.

Further illustrated in Table 8, there was an association between age and arthritis among Guyanese (AOR = 1.13, *p* < 0.001), Jamaicans (AOR = 1.09, *p* < 0.001) and US Caribbean descendants (AOR = 1.08, *p* < 0.001). In all countries examined, there were greater odds for this condition as participants aged. Guyanese females additionally had greater odds (AOR = 1.79, *p* < 0.05) for arthritis. The data further revealed an increased odds for arthritis among Caribbean descendants who had lived in the United States for over 20 years (AOR = 1.94, *p* < 0.05) as well as third generation Caribbean descendants (AOR = 17.26, *p* < 0.001). This increase was also found among US Caribbeans that were not in the labor force (AOR = 1.77, *p* = 0.052). However, the odds for this condition reduced among high school participants within this group (AOR = 0.35, *p* < 0.01).

**Table 8:** Factors associated with lifetime prevalence of arthritis by country.

Characteristics	Guyana	Jamaica	US Caribbeans
	AOR (CI)	AOR (CI)	AOR (CI)
Age	1.13 (1.10–1.15) ^***^	1.09 (1.06–1.12) ^***^	1.08 (1.05–1.11) ^***^
Gender			
Male	1	1	1
Female	1.79 (1.00–3.20) ^*^	1.06 (0.57–2.00)	1.49 (0.89–2.48)
Marital Status			
Never Married	1	1	1
Married	1.83 (0.74–4.52)	0.49 (0.23–1.08)	1.43 (0.57–3.60)
Partnered	1.50 (0.46–4.91)	0.14 (0.02–1.08)	0.74 (0.21–2.55)
Sep-Div-Widow	0.86 (0.34–2.21)	0.43 (0.18–1.04)	1.70 (0.58–4.89)
Education			
Primary/Some HS	1	1	1
High School Grad	1.04 (0.46–2.39)	1.08 (0.39–2.99)	0.35 (0.18–0.68) ^**^
College-Voc-Tech	0.37 (0.14–1.05)	0.94 (0.25–3.54)	0.57 (0.29–1.09)
Employment Status			
Employed	1	1	1
Unemployed	0.30 (0.09–1.04)	1.54 (0.58–4.15)	1.59 (0.38–6.67)
Not in Labor Force	0.79 (0.39–1.60)	1.50 (0.65–3.50)	1.77 (1.02–3.07) ^*^
Equivalized Income			
Bottom Quintile	1	1	1
Second Quintile	1.30 (0.63–2.69)	1.60 (0.75–3.40)	0.58 (0.14–2.34)
Middle Quintile	1.71 (0.67–4.36)	n/a	1.55 (0.67–3.54)
Fourth Quintile	0.78 (0.26–2.31)	1.02 (0.30–3.43)	0.46 (0.19–1.11)
Highest Quintile	0.68 (0.19–2.50)	0.34 (0.04–2.94)	0.67 (0.37–1.20)
Ancestry			
<20 years	--	--	1
>20 years	--	--	1.94 (1.01–3.72) ^*^
Second Generation	--	--	1.94 (0.63–6.04)
Third Generation	--	--	17.26 (7.04–42.33) ^***^

Notes: “AOR” refers to Adjusted Odds Ratio and “n/a” indicates there was insufficient data. ^*^ *p* < 0.05; ^**^ *p* < 0.01; ^***^ *p* < 0.001.

Table 9 shows that across regions, age was related to fair or poor self-rated dental health. For instance, among participants in Guyana (AOR = 1.05, *p* < 0.001) Jamaica (AOR = 1.05, *p* < 0.001) and US Caribbeans (AOR = 1.03, *p* < 0.01), there were increased odds for fair or poor dental health among older participants. This increase was also found among unemployed Guyanese (AOR = 1.57, *p* = 0.05). The data further revealed increased odds for fair or poor dental health among third generation US Caribbeans (AOR = 3.15, *p* < 0.05), as well as middle (AOR = 2.32, *p* < 0.05) and fourth income quintile (AOR = 2.33,* p* < 0.05) participants within this population. Contrasting this finding, however, was the reduced odds for fair or poor dental health among married (AOR = .64, *p* < 0.01) Guyanese participants. Both high school (AOR = 0.67, *p* < 0.01) and college educated (AOR = 0.41, *p* = 0.001) participants within this population also had lower odds for fair or poor self-rated dental health. Lower odds (AOR = 0.40, *p* < 0.01) for fair or poor dental health was also apparent among college educated Jamaicans.

**Table 9:** Factors associated with self-rated dental health (fair/poor) by country.

Characteristics	Guyana	Jamaica	US Caribbeans
	AOR (CI)	AOR (CI)	AOR (CI)
Age	1.05 (1.04–1.06) ^***^	1.05 (1.04–1.06) ^***^	1.03 (1.01–1.05) ^**^
Gender			
Male	1	1	1
Female	0.89 (0.70–1.15)	0.93 (0.676–1.28)	1.19 (0.84–1.68)
Marital Status			
Never married	1	1	1
Married	0.64 (0.46–0.88) ^**^	0.80 (0.54–1.18)	0.89 (0.44–1.82)
Partnered	1.37 (0.96–1.96)	0.79 (0.48–1.27)	1.49 (0.77–2.89)
Sep-Div-Widow	0.89 (0.61–1.29)	0.88 (0.53–1.48)	1.01 (0.52–1.95)
Education			
Primary/Some HS	1	1	1
High School Grad	0.67 (0.49–0.92) ^**^	0.80 (0.50–1.28)	0.52 (0.24–1.12)
College-Voc-Tech	0.41 (0.28–0.62) ^***^	0.40 (0.22–0.72) ^**^	0.61 (0.36–1.05)
Employment Status			
Employed	1	1	1
Unemployed	1.57 (1.08–2.30) ^*^	1.52 (0.98–2.35)	1.85 (0.71–4.83)
Not in Labor Force	0.917 (0.68–1.23)	1.14 (0.79–1.66)	1.32 (0.66–2.63)
Equivalized Income			
Bottom Quintile	1	1	1
Second Quintile	0.99 (0.70–1.39)	0.83 (0.54–1.27)	1.03 (0.59–1.81)
Middle Quintile	0.82 (0.55–1.22)	0.38 (0.10–1.50)	2.32 (1.07–5.04) ^*^
Fourth Quintile	0.99 (0.66–1.51)	0.89 (0.51–1.56)	2.33 (1.02–5.31) ^*^
Highest Quintile	1.02 (0.62–1.66)	0.83 (0.42–1.60)	1.20 (0.60–2.42)
Ancestry			
<20 years	---	---	1
>20 years	---	---	1.42 (0.64–3.14)
Second Generation	---	---	1.27 (0.71–2.26)
Third Generation	---	---	3.15 (1.55–6.40) ^**^

Notes: “AOR” refers to Adjusted Odds Ratio and “n/a” indicates there was insufficient data. ^*^ *p* < 0.05; ^**^ *p* < 0.01; ^***^ *p* < 0.001.

The final analysis shown in Table 10, revealed increased odds for fair or poor general physical health among older Guyanese (AOR = 1.05, *p* < 0.001) and Jamaicans (AOR = 1.03,* p* < 0.001). A similar direction in fair or poor general health was observed for females (AOR = 1.44, *p* < 0.05) and unemployed (AOR = 1.70,* p* < 0.01) Jamaicans. Increased odds for fair or poor self-rated health was also found among US Caribbean (AOR = 2.33, *p* < 0.01) participants who were not in the labor force. This increase odds in fair or poor health was also observed for Caribbean descendant who had lived in the United States for more than 20 years (AOR = 1.92, *p* < 0.05) and among second (AOR = 2.41, *p* < 0.05) and third generation (AOR = 2.48, *p* < 0.01) participants. Contrary to these findings, college educated Guyanese (AOR = 0.50, *p* < 0.01) and Jamaicans (AOR = 0.48, *p* < 0.01) had reduced odds of fair or poor health. 

**Table 10:** Factors associated with self-rated general health (fair/poor) by country.

Characteristics	Guyana	Jamaica	US Caribbeans
	AOR (CI)	AOR (CI)	AOR (CI)
Age	1.05 (1.04–1.06) ^***^	1.03 (1.02–1.04) ^***^	1.02 (0.99–1.04)
Gender			
Male	1	1	1
Female	1.10 (0.84–1.43)	1.44 (1.04–1.98) ^*^	1.57 (0.79–3.10)
Marital Status			
Never married	1	1	1
Married	0.88 (0.63–1.23)	0.87 (0.59–1.28)	0.54 (0.31–0.95) ^*^
Partnered	0.96 (0.64–1.46)	1.30 (0.84–2.01)	1.10 (0.37–3.23)
Sep-Div-Widow	0.95 (0.64–1.40)	1.12 (0.69–1.83)	0.44 (0.16–1.19)
Education			
Primary/Some HS	1	1	1
High School Grad	0.87 (0.62–1.22)	0.69 (0.44–1.09)	0.39 (0.16–0.97) ^*^
College-Voc-Tech	0.50 (0.33–0.774) ^**^	0.48 (0.27–0.83) ^**^	0.37 (0.16–0.89) ^*^
Employment Status			
Employed	1	1	1
Unemployed	1.14 (0.75–1.75)	1.70 (1.11–2.60) ^**^	1.17 (0.62–2.22)
Not in Labor Force	1.22 (0.90–1.66)	1.27 (0.89–1.82)	2.33 (1.28–4.24) ^**^
Equivalized Income			
Bottom Quintile	1	1	1
Second Quintile	0.99 (0.69–1.42)	1.01 (0.66–1.51)	0.78 (0.32–1.93)
Middle Quintile	0.97 (0.64–1.47)	0.42 (0.09–1.99)	1.80 (0.74–4.41)
Fourth Quintile	0.82 (0.52–1.29)	1.10 (0.64–1.91)	1.34 (0.47–3.82)
Highest Quintile	0.90 (0.53–1.54)	0.99 (0.52–1.86)	0.50 (0.25–0.98) ^*^
Ancestry			
<20 years	---	---	1
>20 years	---	---	1.92 (1.07–3.46) ^*^
Second Generation	---	---	2.41 (1.17–4.94) ^*^
Third Generation	---	---	2.48 (1.30–4.72) ^**^

Notes: “AOR” refers to Adjusted Odds Ratio and “n/a” indicates there was insufficient data. ^*^ *p* < 0.05; ^**^ *p* < 0.01; ^***^ *p* < 0.001.

This was also the case for high school (AOR = 0.39, *p* < 0.05) and college educated (AOR = 0.37, *p* < 0.05) US Caribbeans. In addition, lower odds for fair or poor physical health was found among US Caribbeans in the highest income quintile category (AOR = 0.51, *p* < 0.05) within this population.

### 3.3. Discussion

The results of this study revealed that national and cultural residential context influenced health conditions [52]. Negative mental health conditions were generally higher among Caribbean descendants within the United States compared to those found in Caribbean regions. This was particularly evident for alcohol abuse, drug abuse, substance abuse and major depression. For physical conditions, such as diabetes, hypertension and arthritis, a similar pattern was observed. Among Caribbean descendants within the United States, lower fair or poor self-rated health ratings were found compared to individuals in other Caribbean regions. Although the standard of living may be higher within the US context and individuals generally have better access to health care and health resources, in their attempts to achieve and maintain certain lifestyles, Caribbean migrants may be exposing themselves to stressors resulting from poor social and living conditions that may contribute to deterioration in health.

Differences in substance use and mental disorders and physical health in Caribbean countries (e.g., Jamaica* vs.* Guyana) were found. While Guyanese showed higher rates of substance use and mental health disorders, Jamaicans were more inclined to poorer physical health. These differences between Caribbean countries may reflect various factors including higher levels of religious identification and engagement that have been linked to positive self-efficacy, serving as a protective factor against negative mental health conditions, particularly among Jamaicans [53]. In Guyana, social conditions and the lack of resources devoted to mental health care could partly account for this result.

Multivariate analyses also provided added explanation on the influence of context to health in the United States where longer exposure was associated with poorer mental and physical health among Caribbean descendants. First generation Caribbean descendants who had spent over 20 years in the United States and second and third generation participants had increased chances of substance use and mental disorders (e.g., alcohol abuse, drug abuse, substance abuse, depression) and physical (e.g., hypertension, arthritis, dental health, general physical health) outcomes; many of these may be a result of stressful life conditions. These findings lend support to structural theory that substance use and other mental and physical conditions may develop from stressful life circumstances that immigrants encounter the longer they are in host countries [47,54]. These study findings may also be partly attributed to behaviors learned after extended periods in the host country, as immigrant individuals become more assimilated into the wider culture. Over these years, they may embrace social and cultural norms that are more permissive toward certain behaviors and practices that can negatively affect health outcomes more so than in their homelands.

While some conditions found in this study are expected with an increase in age, there were other social and demographic factors that influenced health outcomes across regions. For example, women were less likely to use drugs, alcohol, and other substances. The lack of drug use among Caribbean women may be attributed to strong cultural views and stigmas about the use of substances [55]. Additionally, some Caribbean descendants may associate drug use with weakness and quick fixes to their problems [56]. Socially acceptable gender appropriate behavior may further contribute to this result. For example, women’s display of femininity is an important basis for their social value. Drug, cigarette and alcohol use are viewed as masculine behaviors that women should avoid.

Consistently, age was predictive of more negative physical health. Chances for conditions such as arthritis increase with age. Even though there is greater possibility for diabetes and hypertension as individuals’ age, the accumulation of poor diet and stressful environmental circumstances may also increase the likelihood of these conditions. Added stressful and poor economic circumstances within these regions may further increase the likelihood for conditions such as hypertension. Although it was not examined in this study, it is possible that poor health conditions among aging immigrant groups can result because of fewer social networks [57].

There were several limitations of this study that should be considered when interpreting the results. Because of sample size issues we were unable to explore some health conditions, such as mania, in multivariate analyses. Also, due to small sample sizes, high odds ratios and excessively wide confidence internals should be interpreted with caution; and we were unable to conduct other potentially clarifying analyses that could have assisted in our understanding of the health of immigrant and non-immigrant Caribbean groups. In addition to sample size issues, the study was limited by the lack of a direct measure of acculturation. Even so, a vast majority of immigrant studies using large-scale surveys have used other measures of acculturation, including length of residence or language fluency or acquisition [58,59]. In this study we used length of residency; a disproportionate number of participants within the NSAL hailed from English speaking countries. Similarly, English is the national language in Jamaica and Guyana. The datasets in Caribbean regions were also limited by conditions that are on the rise in Caribbean regions (e.g., obesity) that affect other health outcomes. Moreover, data collected in Jamaica were limited to the greater Kingston Metropolitan Region and should not be generalized to other parts of the country; though a larger percent of the population resides in these areas. Additionally, while there are benefits to using structured clinical assessments (e.g., CIDI), differences in cross-cultural interpretation of concepts could have possibly influenced the study results [60,61,62,63]. Finally, given the narrow focus of the paper we did not include social and contextual variables in models that certainly would have had effects on health conditions (e.g., discrimination, violence,* etc.*).

Notwithstanding these limitations, the study makes many contributions to the literature. Specifically, this is one of few studies to use datasets with probability sampling strategies to address the health disposition of Caribbean descendants across these three geographic locations permitting the results to be generalized to the larger populations from where the samples were drawn. The analysis of these datasets also permits inferences about context and process of migration and generation status on the health of Caribbeans outside the Caribbean region. The use of structured clinical assessments in samples made possible more precise evaluations of mental health on individuals in Caribbean regions, another benefit to this study that has rarely been done before. Finally, the analysis provides support of the advantaged health standing of Caribbean descendants living in Caribbean regions compared to those who have migrated to the US.

Even with the positive benefits of this research, it is important that we continue to move the discourse on the health of immigrant groups by giving greater consideration to other factors, such as the lack of social ties that may increase the risk for substances and other negative mental and physical health conditions, particularly among more acculturated (e.g., 2nd and 3rd generation) groups. In addition to these explorations, a number of questions need to be addressed in future studies and in the coming years with expanding and diverse Caribbean population across regions. Among these questions are: (1) how does the health of Caribbeans differ along ethnic/racial lines?; (2) what are other factors that may account for the poor health conditions of Caribbean residing in the US and other Caribbean regions?; (3) what role do social stressors (e.g., discrimination, violence) play in the health of individuals in Caribbean countries?; (4) what are factors associated with good health for Caribbean descendants within Caribbean regions and other parts of the world?; and (5) what will become of the health of individuals residing in Caribbean countries as they become more adapted to Westernized ideals and lifestyles? With growing consumer economies experiencing an increase in fast food chains and consumptions of processed foods which lack the nutrients and health benefits of natural foods produced in the Caribbean, along with increasing poverty rates within the region, it remains to be seen whether individuals in Caribbean countries will maintain their advantaged health standing over migrated individuals.

## 4. Conclusions

The results of this study highlight the importance of national context in both mental and physical health for Black Caribbeans. These findings were consistent with prior studies that show that longer exposure to the US context is associated with poorer health outcomes for immigrant and immigrant ancestry groups, with the best health outcomes reported for first generation immigrants [2,5,6,9,10,11,16]. This is also the first study of which we are aware that compared the health of US-based probability samples of Caribbeans and probability samples drawn from Jamaica and Guyana. While there is a small but growing literature based upon cross-Caribbean studies of physical health, there are fewer studies on substance use and mental health. Findings from those analyses suggested that the physical health of Caribbean people in the US was worse than the physical health of those in Jamaica and Guyana. Furthermore, the mental health (major depression) of people in Guyana was worse than their counterparts in Jamaica. On the other hand, the physical health outcomes for Jamaicans were generally worse than those for persons in Guyana. Overall, these findings suggest crucial interrelationships between context and health and offer important avenues for future research on the mechanisms that may undergird these associations.

## Human Protection

Human subjects approval for the NSAL was obtained through the University of Michigan’s institutional review board. For the Jamaica and Guyana NSAL supplements, after approval was granted by the Ministry of Health in both countries, this was used to gain IRB approval at the University of Michigan and for foreign clearance from the NIH and State Department.
